# The role of domestic animals in the epidemiology of human african trypanosomiasis in Ngorongoro conservation area, Tanzania

**DOI:** 10.1186/s13071-015-1125-6

**Published:** 2015-10-06

**Authors:** Juan P. Ruiz, Hamisi S. Nyingilili, Geofrey H. Mbata, Imna I. Malele

**Affiliations:** Fulbright Student, Fulbright Institute of International Education, 809 United Nations Plaza, New York, 10017 NY USA; Vector & Vector Borne Diseases Research Institute, Majani Mapana, Off Korogwe Road, Box 1026, TANGA, TANZANIA

**Keywords:** Trypanosomiasis, Goats, Sheep, Tanzania, T.b. rhodesiense, Sleeping sickness, HAT, AAT, PCR, LAMP

## Abstract

**Background:**

Trypanosomiasis is a neglected tropical disease caused by the trypanosome parasite and transmitted by the tsetse fly vector. In Sub-saharan Africa, both the human and animal variants of the disease are a great obstacle towards agriculture, development, and health. In order to better understand and therefore combat Trypanosomiasis, characterizing disease hotspots across species is critical.

**Methods:**

In this study, 193 samples from cattle, sheep, and goats were collected from eight sites. Samples were taken from animals belonging mostly to Maasai herdsmen in the Ngorongoro Crater Conservation Area (NCA) and analysed for the presence of trypanosomiasis infection using PCR techniques. Those that tested positive for *T. brucei* parasite were further tested using SRA LAMP technique to check for *T. brucei rhodesiense,* the human infective subspecies of parasite.

**Results:**

Our study found a high incidence of *Trypanosoma brucei* infections across species. Of animals tested, 47 % of cattle, 91.7 % of sheep, and 60.8 % of goats were infected. Most of the infections were of the *T. brucei* species. We also identified sheep and goats as carriers of the *T. brucei rhodesiense* subspecies, which causes acute human trypanosomiasis.

**Conclusions:**

Together, these results point toward the need for stricter control strategies in the area to prevent disease outbreak.

## Background

Tsetse flies (*Glossina)* are one of the insect vectors that have most greatly affected human and animal populations in Tanzania, as they transmit the blood-borne flagellate protozoa known as the Trypanosome parasite. The diseases caused by trypanosomes are Human African Trypanosomiasis (HAT or Sleeping Sickness) and Animal African Trypanosomiasis (AAT or Nagana). Trypanosomiasis and tsetse flies remain the greatest obstacles to livestock development in Africa [1]. The wide occurrence of trypanosomiasis in areas of tsetse habitation [2] has effectively prevented the establishment of sustainable development of viable agricultural systems in many areas of great agricultural potential [3].

In Tanzania, tsetse flies infest approximately 33 % of the 940,027 sq km of the mainland and have been a limitation to the country’s development [4, 5]. These parasites cause sub-Saharan Africa about $4.75 billion USD of loss in agricultural potential due to livestock losses to AAT. It is estimated that productivity in livestock is heavily reduced by this disease: 70 % reduction of cattle density, 50 % in sale of meat and milk, 20 % in calving rates, and increases in calf mortality by 20 % [1].

Due to recent changes in habitat because of deforestation, climate change, and loss of native blood meal species, domestic animals, particularly livestock, are becoming an important source of food for tsetse flies [6]. In areas where there is a high density of human, cattle, and fly cohabitation, the risk of disease transmission across species is high, as it is estimated that sleeping sickness is more likely to be transmitted by a cattle-fly-human transmission cycle than a human-fly-human cycle [7]. In order to prevent large outbreaks of HAT, it is critical to identify reservoirs of HAT within cattle populations and to implement appropriate control strategies, whether at the parasite or vector level [8]. In Tanzania, the Ngorongoro Conservation Area (NCA) is one of the most popular tourist attractions and is bordered by Serengeti National Park, Maswa Game Reserve and Loliondo Game Reserve, all areas which are tsetse infested and at risk of HAT. NCA practices multiple land use whereby wildlife, livestock and people co-exist. The large pastoral Maasai population which lives and herds cattle in this area is therefore at great risk.

The ability to identify different species of trypanosomes has evolved from pure microscope observation [9] and unreliable field diagnostic techniques with low sensitivity to faster and more reliable gene sequencing techniques. These included both species-specific DNA probes [10–12], as well as species-specific Polymerase Chain Reaction (PCR) tests [13–15]. The newest methods use generic primers to sequence highly conserved regions across trypanosome species in between which are regions of varying length. Of these methods, one takes advantage of the interspecies size variation of the PCR product of the ITS-1 ribosomal RNA region [16–18]. The other technique uses fluorescent primers to detect length variations in multiple fragments in 18S and 28S ribosomal RNA regions [19, 20]. This allows for quick and specific parasite recognition without the use of various PCR tests, as was the case with the species-specific primers. It also allows for the quick detection of multiple infections within the same host. Using this last technique, our laboratory has previously detected a new species of the subgenus *Trypanozoon* [20]. In this study, samples from the NCA were analysed for trypanosome infection and further tested for presence of human infective *T. brucei rhodesiense.*

## Methods

### Ethical approval

Approval for this research project was granted by the Tanzania Commission for Science and Technology (COSTECH).

### Data collection

**Fig. 1:**
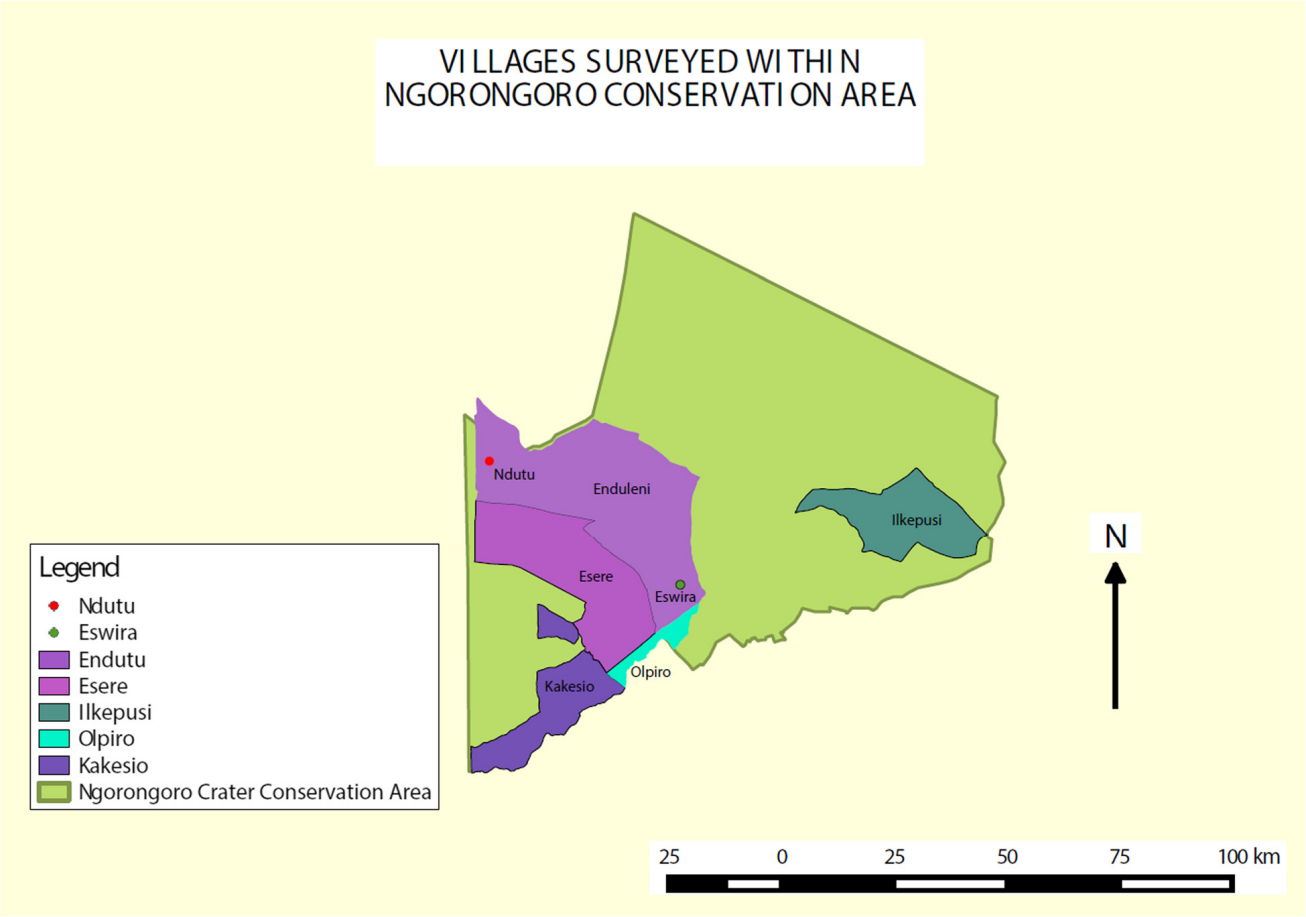
Geographic distribution of sites sampled within NCA

Samples were collected twice, once in November 2013 and again in February 2014, both times before the onset of the rainy season from eight sites (Table 1; Fig. 1). Briefly, herdsmen were alerted one day earlier. We requested for their consent to sample their animals. Because most pastoralists in the area are familiar with the disease, they easily agreed to have their animals sampled. A small puncture was done on the peripheral ear vein and blood collected in duplicate heparinised capillary tubes for further processing back in the field station. Most samples were taken in the morning, before cattle were taken out to graze.

**Table 1:** Table of sites within NCA sampled, time of sampling, sample species collected and infection prevalence at each site

Site	Season Tested	Bovine Samples	Caprine Samples	Ovine Samples	Total Samples	Infection Prevalence at Site	95 % Confidence Interval
Kakesio	Nov-13	20	14	6	40	22.50 %	12.3 % - 37.5 %
Enduleni	Nov-13	12	0	0	12	16.70 %	4.7 % - 44.8 %
Esere	Nov-13	8	6	2	16	56.25 %	33.2 % - 76.9 %
Ndutu	Nov-13	18	0	0	18	44.44 %	24.6 % - 66.3 %
Nasporeo	Nov-13	0	0	10	10	100 %	72.2 % - 100 %
Olpiro	Feb-14	6	17	0	23	87 %	67.9 % - 95.5 %
Eswira	Feb-14	18	12	0	30	60 %	42.3 % - 75.4 %
Ilkepusi	Feb-14	29	0	2	31	71 %	53.4 % - 83.9 %
Enduleni	Feb-14	6	6	0	12	83.33 %	55.2 % - 95.3 %

### Sample storage

Capillary tubes were spun centrifuged at 12,000 rpm for about 5 min in Hawksley® centrifuge machine (Hawksley Ltd, Sussex, UK). Once proper separation of blood had taken place, the buffy coat only was spotted onto Whatman FTA cards for sample storage. Previous studies have shown this to be the most convenient and efficient method for collecting parasite DNA [21–25]. All samples were then transported to the lab and stored until all samples had been collected.

### DNA extraction

DNA extraction from Whatman FTA cards and filter paper have been previously described [21, 22]. Briefly, 10 punches of 0.1 mm were taken from each card and pooled. DNA was then eluted using Chelex®100 20 % solution at 55 °C for 60 min and then briefly vortexed before boiling for 10 min.

### qPCR and LAMP

DNA was then tested for the presence of *Trypanosome* DNA. Primers for the conserved ITS region (purchased from Bionner ISO 9001:2000) were used as previously described [17, 26]. PCR reactions were carried out using Phusion PCR kit (Thermo Fischer F-530S).

Those samples that tested positive for *T.brucei* were tested using the SRA LAMP technique as previously described [27] to determine whether they have the human resistance associated (SRA) gene uniquely expressed by *T. b. rhodesiense* (SRA-LAMP) [28, 29]. In this technique samples were considered positive if they turned green when Sybergreen was added. Those which never changed colour were assumed to contain samples that were SRA negative and were thus considered to contain *T. brucei* only. In this technique distilled water was used as a negative control and *T. b. rhodesiense* DNA, kindly donated by Dr. Enock Matovu (Makerere University, Kampala), was used as a positive control.

### Statistical analysis

95 % confidence intervals for infection prevalence within populations sampled were calculated using the Wilson method for calculating 95 % confidence intervals of binomial populations [30]. Briefly, the populations sampled were assumed to follow a binomial distribution in which two outcomes were possible: either the samples tested positive (success) or negative (failure). The Wilson method was then applied: the total number of samples within a given subpopulation represented the total number of trials and the number of positive samples represented the number of successes. For example, in Table 1, the site of Kakesio was considered to be an independent set of 40 trials (*n* = 40) with 9 successes (x = 9). In Table 2, as another example, the adult bovine population was considered an independent set of 70 trials (*n* = 70) with 38 successes (x = 38). Note that for these calculations, each subpopulation was treated as an independent set of trials. Because of this, the “tightest” confidence intervals come from those calculated for all species in the bovine, ovine, or caprine populations, and these represent the prevalence of infections in those species throughout the area. All calculations were carried out using R statistics software with the “binom” package.

**Table 2:** Table of infection prevalence separated by species. Note the same samples are being categorized differently on the top and bottom of the table according to age or sex

Species	Age	Total	Infected	Prevalence	95 % Confidence Interval
Bovine	Adult	70	38	54.30 %	42.7 % - 65.4 %
Bovine	Young	47	17	36.20 %	24 % - 50.5 %
Caprine	Adult	35	23	65.70 %	49.1 % - 79.2 %
Caprine	Young	16	8	50 %	28 % - 72 %
Ovine	Adult	15	15	100 %	79.6 % - 100 %
Ovine	Young	9	7	77.80 %	45.3 % - 93.7 %
Species	Sex	Total	Infected	Prevalence	95 % Confidence Interval
Bovine	Female	82	41	50 %	39.4 % - 60.6 %
Bovine	Male	35	14	40 %	25.6 % - 56.4 %
Caprine	Female	33	20	60.60 %	43.7 % - 75.3 %
Caprine	Male	18	11	61.10 %	38.6 % - 79.7 %
Ovine	Female	14	13	92.90 %	68.5 % - 98.7 %
Ovine	Male	10	9	90 %	59.6 % - 98.2 %
Species		Total	Total Infected	Prevalence	95 % Confidence Interval
Bovine		117	55	47 %	38.2 % - 56 %
Caprine		51	31	60.80 %	47.1 % - 73 %
Ovine		24	22	91.70 %	74.2 % - 97.7 %

## Results

As can be seen in Tables 1 and 2, the majority of the samples were taken from Bovine specimens. This can be attributed to the fact that most of the herdsmen visited owned more cattle than goats and sheep, but were also more readily available and willing for us to sample their cows.

**Fig. 2:**
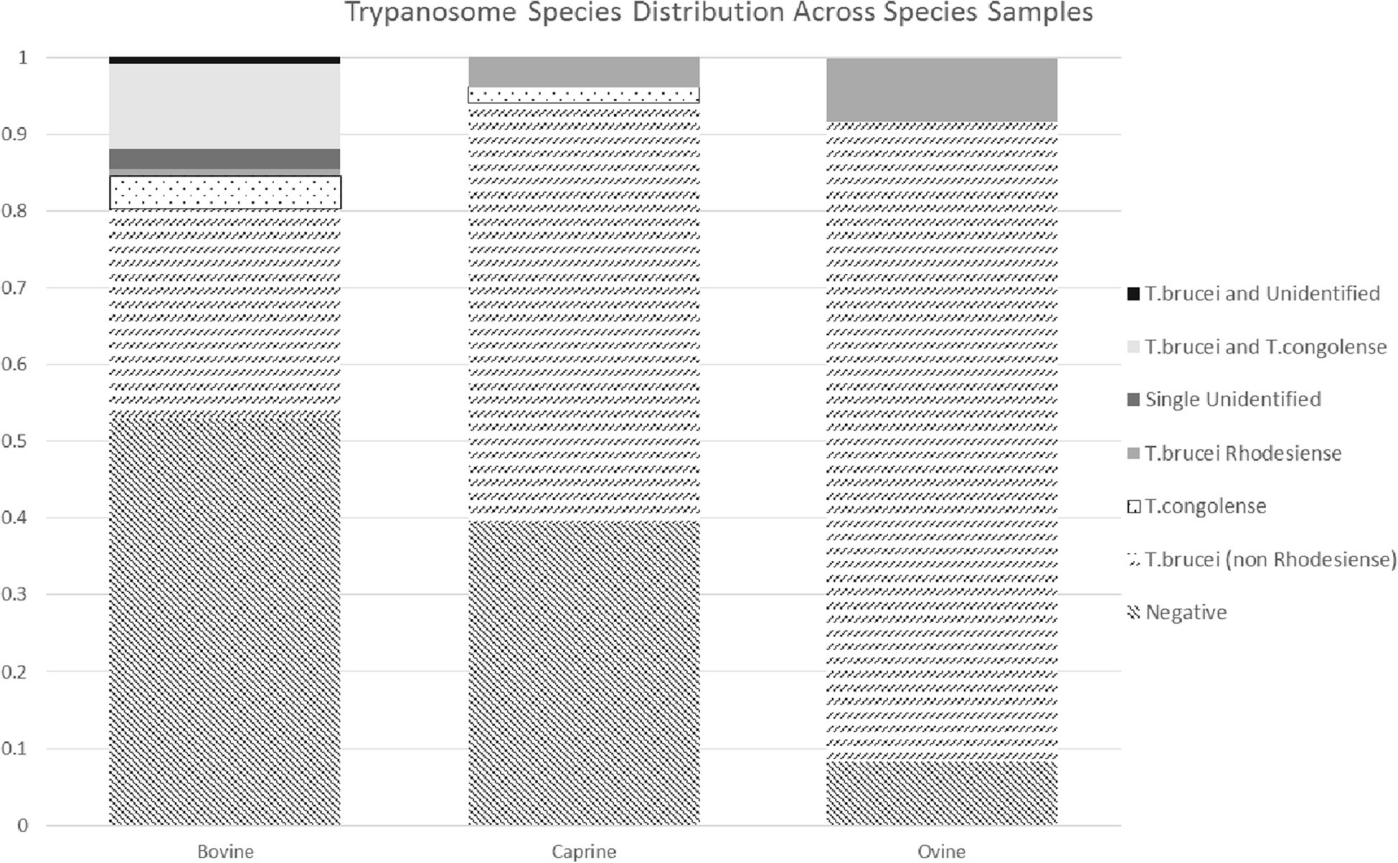
Trypanosome species distribution within each host species sampled

Of those samples testing positive for parasite DNA, the majority, 85 out of 108, were infected by a single trypanosome, *T. brucei* (Table 3, Fig. 2). Double infections were less rare (14). They were strictly limited to bovine hosts and all but one were *T. brucei* and *T. congolense*. Only 9 samples were single infections due to parasites other than *T. brucei*. For the *T. brucei* infections tested for *T. brucei rhodesiense*, only 5 out of the 85 tested positively (Table 3).

**Table 3:** Table of trypanosome species detected within each host species sample set

Species	*T. brucei (non rhodesiense)*	*T. brucei rhodesiense*	*T. congolense*	*Single Unidentified*	*T. brucei and T. congolense*	*T. brucei and Unidentified*	*Negative*	Total Sampled
Bovine	32	1	5	3	13	1	62	117
Caprine	28	2	1	0	0	0	20	51
Ovine	20	2	0	0	0	0	2	24

## Conclusion

This is the first study that reports on the role of ovine and caprine species in the transmission of *T.brucei rhodesiense* in Tanzania. While both cattle and more recently, pigs have been reported as potential reservoirs of acute human sleeping sickness [31, 32] this is the first study to address this in small ruminants. These findings, along with our discovery of a high incidence of animals infected with trypanosomiasis in the area, call for a shift in the care of livestock among the Maasai herdsmen. Normally, cattle are given the required attention and treated accordingly once animals become symptomatic. Small ruminants, even when symptomatic, are not given the required treatments. In areas of tsetse infestation, vector control and treatment of diseased animals should be common practice. For the prevention of outbreaks of the human version of the disease, these should be coupled with periodic screening of patients in areas where humans, domestic animals, wild animals, and tsetse flies cohabitate.
